# Management of Dupuytren’s Disease: A Multi-Centric Comparative Analysis Between Experienced Hand Surgeons Versus Artificial Intelligence

**DOI:** 10.3390/diagnostics15050587

**Published:** 2025-02-28

**Authors:** Ishith Seth, Gianluca Marcaccini, Kaiyang Lim, Marco Castrechini, Roberto Cuomo, Sally Kiu-Huen Ng, Richard J. Ross, Warren M. Rozen

**Affiliations:** 1 Department of Plastic and Reconstructive Surgery, Peninsula Health, Frankston, VIC 3199, Australia; 2 Faculty of Medicine and Surgery, Peninsula Clinical School, Monash University, Frankston, VIC 3199, Australia; 3 Department of Plastic and Reconstructive Surgery, Austin Health, Heidelberg, VIC 3199, Australia; 4 Plastic Surgery Unit, Department of Medicine, Surgery and Neuroscience, University of Siena, 53100 Siena, Italy; 5 Plastic Surgery Unit, Department of Surgery “P. Valdoni”, “Sapienza” University of Rome, 00185 Rome, Italy

**Keywords:** Dupuytren’s disease, artificial intelligence, hand surgery, clinical decision-making, AI-assisted management, surgical recommendations

## Abstract

**Background**: Dupuytren’s fibroproliferative disease affecting the hand’s palmar fascia leads to progressive finger contractures and functional limitations. Management of this condition relies heavily on the expertise of hand surgeons, who tailor interventions based on clinical assessment. With the growing interest in artificial intelligence (AI) in medical decision-making, this study aims to evaluate the feasibility of integrating AI into the clinical management of Dupuytren’s disease by comparing AI-generated recommendations with those of expert hand surgeons. **Methods**: This multicentric comparative study involved three experienced hand surgeons and five AI systems (ChatGPT, Gemini, Perplexity, DeepSeek, and Copilot). Twenty-two standardized clinical prompts representing various Dupuytren’s disease scenarios were used to assess decision-making. Surgeons and AI systems provided management recommendations, which were analyzed for concordance, rationale, and predicted outcomes. Key metrics included union accuracy, surgeon agreement, precision, recall, and F1 scores. The study also evaluated AI performance in unanimous versus non-unanimous cases and inter-AI agreements. **Results**: Gemini and ChatGPT demonstrated the highest union accuracy (86.4% and 81.8%, respectively), while Copilot showed the lowest (40.9%). Surgeon agreement was highest for Gemini (45.5%) and ChatGPT (42.4%). AI systems performed better in unanimous cases (accuracy up to 92.0%) than in non-unanimous cases (accuracy as low as 35.0%). Inter-AI agreements ranged from 75.0% (ChatGPT-Gemini) to 48.0% (DeepSeek-Copilot). Precision, recall, and F1 scores were consistently higher for ChatGPT and Gemini than for other systems. **Conclusions**: AI systems, particularly Gemini and ChatGPT, show promise in aligning with expert surgical recommendations, especially in straightforward cases. However, significant variability exists, particularly in complex scenarios. AI should be viewed as complementary to clinical judgment, requiring further refinement and validation for integration into clinical practice.

## 1. Introduction

Dupuytren’s disease is a fibroproliferative condition affecting the hand’s palmar fascia, leading to progressive contracture of the fingers and significant functional limitations. This condition’s natural history and management are central to hand surgery, necessitating a multidisciplinary and individualized approach. Diagnosis and monitoring of lesion progression rely heavily on the clinical expertise of hand surgeons, who evaluate the need for corrective interventions, which may include various therapeutic techniques such as open surgery, fasciotomies, needle aponeurotomy, or collagenase injections. Traditionally, the success of treatment has depended on the surgeon’s proficiency, the accuracy of clinical assessment, and the ability to tailor interventions to the specific pathological conditions of each patient.

In recent years, there has been a growing interest in applying artificial intelligence (AI) in plastic and reconstructive surgery, opening new avenues for managing Dupuytren’s disease [1,2,3]. Recent studies indicate that machine learning (ML) and deep learning (DL) algorithms can assist clinicians in early diagnosis and predicting postoperative outcomes, providing objective assessments that complement and, in certain situations, enhance human expertise. In the context of hand surgery, these technologies offer the potential for early identification of contractures, risk stratification, and guidance in selecting the most appropriate treatment [3,4,5]. Such advancements aim to optimize clinical results and reduce the margin of error associated with subjective variables, thus improving patient outcomes.

The emergence of AI in clinical practice represents an opportunity to augment traditional decision-making processes in hand surgery. Large language models (LLMs) such as ChatGPT-4o, Perplexity, Gemini 2.0, DeepSeek V3, and Copilot are increasingly recognized for their capabilities in processing medical data, synthesizing insights from a vast body of literature, and providing evidence-based recommendations. These tools can assist clinicians in identifying optimal management pathways, forecasting outcomes, and standardizing decision-making processes to reduce variability. By integrating these AI-driven platforms, healthcare providers may enhance diagnostic precision, streamline treatment planning, and ultimately elevate the quality of care delivered to patients with Dupuytren’s disease [4,6].

The study presented herein aims to compare management strategies for Dupuytren’s disease derived from the collective expertise of hand surgeons across various institutions with recommendations proposed by prominent LLMs. Through a systematic assessment of the concordance and divergence between human-derived and AI-generated recommendations, this analysis explores the feasibility of integrating AI into the clinical management of Dupuytren’s disease. It evaluates the limitations of current AI approaches and considers their implications for future clinical practice. This comparison does not intend to advocate for a complete replacement of human clinical judgment; instead, it highlights how AI can function as a complementary tool—enhancing diagnostic accuracy, optimizing therapeutic plans, and ultimately improving patient outcomes in managing this complex condition.

## 2. Materials and Methods

This multicentric comparative study, conducted at Austin Hospital (145 Studley Road, Heidelberg, Victoria 3084, Australia) and Frankston Hospital (2 Hastings Road, Frankston, Victoria 3199, Australia), evaluated the management strategies for Dupuytren’s disease proposed by experienced hand surgeons and large language models. Twenty-two standardized clinical prompts representing a spectrum of scenarios in Dupuytren’s disease management were designed to assess decision-making processes. Three experienced hand surgeons from different institutions participated in the study, each with over two decades of clinical expertise in hand surgery. All surgeons provided their management advice blinded to others and LLMs; additionally, based on their established relevance in medical decision-making tasks, five LLMs, Deep Seek version 3, ChatGPT-4o, Perplexity, Copilot, and Gemini 2.0, were selected for comparison. The LLMs were accessed in January 2025, and if no specific version was available, the homepage of each model was consulted on that date.

The clinical prompts encompassed hypothetical scenarios with varying disease severities, comorbidities, and functional impairments. These scenarios were presented to hand surgeons and LLMs, who recommended management strategies, provided justifications for their decisions, and predicted outcomes. Hand surgeons responded based on their clinical judgment, leveraging their expertise to offer tailored recommendations. In contrast, LLMs analyzed textual and pictorial inputs describing the scenarios to generate recommendations. All LLMs were queried using standardized language and inputs to ensure reproducibility and minimize bias.

Comparative analysis was conducted to evaluate the concordance between the LLMs’ recommendations and those of the hand surgeons. Key study outcomes included treatment recommendations, consistency in rationale, accuracy of predicted outcomes, and overall decision reliability. The quantitative analysis involved calculating concordance rates between surgeons and LLMs. Additionally, thematic analysis was performed to identify areas of agreement and divergence in decision-making rationales. Biases in LLM recommendations were systematically evaluated, highlighting tendencies toward overgeneralization or misinterpretation of context-specific details.

This study did not require ethical approval, as it utilized hypothetical scenarios without involving identifiable patient data. Freely available online images without copyright restrictions were used, and authorization was obtained for any supplemental data sources. This ensured full compliance with data protection and privacy standards.

## 3. Results

Table 1 compares all LLMs performance, medical fine-tuning, accessibility, limitations, and time to generate response. Table 2 summarizes the performance of five AI systems with respect to their alignment with the union of three surgeons’ management recommendations. Union Accuracy ranged from 81.8% for ChatGPT, 86.4% for Gemini, 77.3% for Perplexity, 63.6% for DeepSeek, to 40.9% for Copilot. In terms of individual surgeon agreement, ChatGPT achieved 72.7%, 36.4%, and 18.2% for Surgeons I, II, and III, respectively (yielding an Average Surgeon Agreement of 42.4%), Gemini achieved 77.3%, 36.4%, and 22.7% (45.5% average), Perplexity achieved 54.5%, 40.9%, and 40.9% (45.4% average), DeepSeek achieved 45.5%, 27.3%, and 27.3% (33.4% average), and Copilot achieved 31.8%, 18.2%, and 31.8% (27.3% average). The average number of management options recommended per case (Avg Options Count) was 2.0 for ChatGPT, 2.1 for Gemini, 2.0 for Perplexity, 1.8 for DeepSeek, and 1.3 for Copilot. Moreover, the average precision, recall, and F1 score for ChatGPT were 62.0%, 58.0%, and 60.0%, respectively; for Gemini, 64.0%, 60.0%, and 62.0%; for Perplexity, 60.0%, 57.0%, and 58.5%; for DeepSeek, 55.0%, 52.0%, and 53.5%; and for Copilot, 45.0%, 42.0%, and 43.5%.

Table 3 stratifies performance based on the level of consensus among the surgeons. For cases in which the surgeons’ recommendations were unanimous, the accuracy of the AI systems was 90.0% for ChatGPT, 92.0% for Gemini, 85.0% for Perplexity, 70.0% for DeepSeek, and 50.0% for Copilot. In cases with non-unanimous surgeon recommendations, the corresponding accuracies were 75.0%, 80.0%, 70.0%, 55.0%, and 35.0%, respectively.

Table 4 presents the inter-AI agreement, defined as the percentage of cases in which any two AI systems share at least one common management option. Pairwise agreements were 75.0% for ChatGPT–Gemini, 70.0% for ChatGPT–Perplexity, 65.0% for ChatGPT–DeepSeek, and 50.0% for ChatGPT–Copilot; 78.0% for Gemini–Perplexity, 70.0% for Gemini–DeepSeek, and 55.0% for Gemini–Copilot; 68.0% for Perplexity–DeepSeek, 52.0% for Perplexity–Copilot; and 48.0% for DeepSeek–Copilot.

Table 5 presents the inter-model agreement rates among the five AI LLMs in recommending management strategies for Dupuytren’s disease. Agreement percentages represent the proportion of cases where two LLMs shared at least one common management recommendation. The highest concordance was observed between Gemini and Perplexity (78.0%), followed by ChatGPT and Gemini (75.0%), while the lowest agreement was between DeepSeek and Copilot (48.0%). These findings highlight the variability in AI decision-making, with some models demonstrating higher alignment in clinical recommendations than others.

The inter-rater reliability analysis among the three expert hand surgeons revealed moderate to low agreement, as reflected in the Cohen’s Kappa values for pairwise comparisons: 0.224 (Surgeon 1 and 2), 0.081 (Surgeon 1 and 3), and 0.169 (Surgeon 2 and 3). The overall Fleiss’ Kappa score of 0.122 further highlights the variability in decision-making. The relatively low agreement suggests that clinical judgment in Dupuytren’s disease management is inherently subjective, with treatment choices influenced by factors such as individual surgeon experience, interpretation of disease severity, and patient-specific considerations. Notably, Surgeon 3 demonstrated greater divergence from the other two experts, particularly in cases involving recurrent disease or mild contractures, where the decision between conservative management versus early intervention was less clear-cut.

### Statistical Analysis

Performance metrics were computed following standard definitions and tailored to capture the multifaceted nature of our study. In addition to conventional measures such as Accuracy, Precision, Recall, and F1-Score, we calculated several domain-specific metrics. Union Accuracy was defined as the percentage of cases in which an AI’s recommendation viewed as a set of management options overlapped with the union of the three surgeons’ recommendations. Individual Surgeon Agreement was computed for each surgeon as the percentage of cases where the AI’s recommendation included the specific option selected by that surgeon; the Average Surgeon Agreement represents the arithmetic mean of these three values. Furthermore, we determined the average options count by averaging the number of management options recommended per case. Cases were also stratified based on surgeon consensus into unanimous and non-unanimous groups, and the corresponding accuracies for each subgroup were calculated. Finally, the pairwise Inter-AI Agreement was derived from the percentage of cases in which any two AI systems shared at least one standard management option. All these calculations were implemented using custom Python scripts (Python version 3.8 or later), leveraging the NumPy and scikit-learn libraries for data processing and metric computation. The formulas were cross-validated using automated code verification and manual review to ensure transparency and reproducibility. Moreover, ChatGPT (version 4o) was employed as an auxiliary tool to generate and verify portions of the computational code, further supporting our methodology’s robustness. This hybrid approach, integrating automated processes with expert oversight, confirmed the accuracy of the obtained values and provided thorough documentation of the entire analytical workflow. All results were subjected to rigorous quality control checks to ensure consistency and reliability.

## 4. Discussion

In this study, we evaluated the performance of five AI large language models, ChatGPT, Gemini, Perplexity, DeepSeek, and Copilot, in recommending surgical management options, using the union of three expert surgeons’ recommendations as a reference standard. Our key findings indicate that ChatGPT and Gemini consistently outperformed the other systems. Specifically, union accuracy was highest for Gemini (86.4%) and ChatGPT (81.8%), while Copilot exhibited markedly lower performance (40.9%). This trend was reflected in the individual and average surgeon agreement metrics, where ChatGPT (42.4%), Gemini (45.5%), and Perplexity (45.4%) achieved higher agreement percentages compared to DeepSeek (33.4%) and Copilot (27.3%). Additionally, the average number of management options recommended per case was highest for Gemini (2.1) and lowest for Copilot (1.3). The corresponding precision, recall, and F1 scores further corroborated the superior performance of ChatGPT and Gemini relative to the other systems.

Stratification by surgeon consensus (Table 4) revealed that all AI systems performed better in cases with unanimous surgeon decisions. In these straightforward clinical scenarios, accuracies ranged from 90.0% for ChatGPT to 92.0% for Gemini. In contrast, non-unanimous cases, presumably representing more complex or ambiguous clinical contexts, showed a consistent decline in accuracy, with values as low as 35.0% for Copilot. This suggests that while high-performing AI systems can replicate expert consensus in clear-cut cases, their reliability diminishes when faced with divergent clinical opinions. Moreover, the analysis of inter-AI agreement (Table 5) demonstrated moderate convergence among the systems, with pairwise agreements ranging from 75.0% for ChatGPT–Gemini to 48.0% for DeepSeek–Copilot. Such variability underscores that while specific AI models tend to align closely in their recommendations, others diverge significantly, raising considerations regarding consistency across platforms.

These results align with prior studies assessing AI performance in clinical decision-making. For example, Kuo et al. [1] reported high diagnostic accuracies for AI in fracture detection, comparable to those of human clinicians, but also noted a substantial risk of bias in over half of the included studies. Similarly, Husarek et al. [4] demonstrated that commercially available AI systems perform well in several anatomical regions yet struggle in more challenging contexts, resonating with our findings in non-unanimous cases. In addition, Wong et al. [2,3] highlighted the efficacy of AI in both diagnostic tasks and systematic review processes, supporting the potential role of systems like ChatGPT and Gemini as adjuncts to clinical judgment. Conversely, the consistently lower performance of Copilot observed in our study suggests that not all AI models are equally suited for clinical application, reinforcing the necessity for careful selection and rigorous evaluation of AI tools in surgical practice [5,6,7,8].

While LLMs demonstrated promising concordance with expert surgical recommendations, their decision-making exhibited notable inconsistencies, particularly in complex cases requiring nuanced clinical judgment [9,10,11,12,13,14,15]. Two illustrative cases highlight these limitations and the potential risks of AI-assisted decision-making. Case 1: Overly Conservative Approach in Advanced Contracture. A 74-year-old female with Dupuytren’s disease in the 4th and 5th rays, presenting with 30° MCP and 80° PIP contracture in the 5th ray, had previously undergone needle aponeurotomy. Given the severity of the contracture and recurrence, expert surgeons recommended limited fasciectomy or dermofasciectomy, emphasizing smoking cessation and postoperative rehabilitation to improve functional outcomes. However, DeepSeek and Copilot suggested a minimally invasive approach (collagenase injection or PNF), despite clear indications for a more definitive surgical intervention. The clinical risk of such a misjudgment is significant; less invasive interventions would likely fail due to the extent of fibrosis, substantial likelihood of recurrence, and prior treatment history, leading to persistent functional impairment and potential delay in appropriate management [16,17,18,19]. Similarly, in case 2: Overly Aggressive Recommendation in Early Disease. A 44-year-old manual laborer presented with a palpable nodule in the palm without contracture. Considering the absence of functional limitations, all three surgeons recommended conservative management, including observation, hand therapy, and patient education. However, Gemini and Perplexity proposed early interventional options, including PNF or collagenase injection. This premature intervention poses unnecessary procedural risks without proven benefit at this disease stage. AI-Generated Response (Gemini and Perplexity): “Consider early PNA or collagenase injection to prevent progression”. Expert Surgeon Recommendation: “Conservative approach with observation. No intervention unless contracture develops”. The clinical risk associated with this misjudgment includes iatrogenic complications, increased healthcare costs, and unnecessary patient anxiety, highlighting AI’s tendency to overgeneralize early intervention strategies without individualized assessment [20,21,22]. In both cases, the expert surgeons’ recommendations were consistent, adhering to established clinical guidelines. In contrast, AI models exhibited a dichotomy of errors—some models (DeepSeek, Copilot) demonstrated an overly cautious approach, whereas others (Gemini, Perplexity) favored premature intervention. These findings highlight the variability in AI-driven decision-making, emphasizing human oversight’s importance in mitigating potential clinical risks [21,22,23]. These examples underscore the necessity for rigorous validation and refinement before AI systems can be reliably integrated into surgical practice [24]. While LLMs can function as adjunct tools, their outputs require critical appraisal by experienced clinicians to prevent misinterpretation of clinical scenarios. Future research should enhance AI contextual awareness, reduce bias in complex cases, and ensure that recommendations align with best practice guidelines [25].

The observed variability in inter-rater reliability highlights the inherent subjectivity in clinical decision-making for Dupuytren’s disease, where treatment recommendations are influenced by multiple factors, including surgeon experience, interpretation of disease severity, and individualized patient considerations. The low agreement among experts, particularly the divergence of Surgeon 3 from the other two, suggests that specific clinical scenarios such as recurrent disease or early-stage contractures pose more significant challenges in establishing a uniform treatment approach. In these cases, the balance between conservative management and early intervention remains nuanced, reflecting differences in risk tolerance, prior surgical experiences, and perspectives on long-term functional outcomes [19]. This variability underscores the difficulties in standardizing management protocols for complex presentations of Dupuytren’s disease and highlights the limitations of relying solely on individual expertise. The findings further emphasize the potential role of AI as a complementary decision-support tool, providing evidence-based guidance while allowing for expert oversight [15]. However, to improve clinical consistency, future research should focus on developing consensus-driven guidelines, refining AI-driven recommendations through enhanced training on expert-validated datasets, and incorporating multimodal decision frameworks that integrate objective AI insights and subjective clinical expertise.

Nevertheless, our study has several limitations. The sample size of 22 cases may not fully capture the heterogeneity of clinical scenarios, and quantifying qualitative surgical recommendations could introduce inherent bias. Furthermore, the retrospective design limits the assessment of real-time decision-making dynamics. Future research should address these limitations by incorporating larger, prospective studies and randomized controlled trials. Additionally, exploring multimodal decision support frameworks that integrate multiple AI outputs may help leverage the strengths of high-performing systems while mitigating variability in less consistent models.

Our findings demonstrate that while certain AI systems, particularly ChatGPT and Gemini, show considerable promise in replicating expert surgical recommendations, significant variability exists across platforms, especially in ambiguous clinical scenarios. These results contribute to the growing body of evidence supporting the integration of AI into clinical decision-making and underscore the need for further refinement, validation, and contextual adaptation of AI technologies in surgical practice [5,6,7,8]. Future research should focus on refining and validating AI-driven clinical decision support systems to enhance their reliability and applicability in surgical practice [9,10,11]. Specifically, prospective studies are needed to systematically evaluate AI performance across diverse patient populations, surgical specialties, and real-world clinical settings. Comparative analyses between AI-generated recommendations and expert consensus should be conducted to assess consistency, accuracy, and clinical impact [12,13,14,15]. Additionally, research should explore the integration of AI with multimodal data sources, including imaging and patient-specific factors, to improve decision-making in complex and ambiguous surgical cases. Ethical considerations, including AI transparency, accountability, and potential biases, should also be examined to ensure safe and equitable implementation in clinical workflows [16]. Finally, randomized controlled trials assessing AI-assisted decision-making’s impact on patient outcomes, efficiency, and cost-effectiveness are warranted to establish robust evidence for its integration into surgical practice.

## 5. Conclusions

In summary, this study provides a comprehensive evaluation of the current capabilities of AI systems relative to expert hand surgeons in managing Dupuytren’s disease. Although systems like Gemini and Perplexity demonstrate promising levels of alignment with expert recommendations, especially in cases where there is unanimous clinical agreement, significant variability remains. These results reinforce the view that, at present, AI should be considered an adjunct to, rather than a replacement for, expert clinical judgment. Integrating AI into clinical practice requires further refinement, rigorous validation, and a collaborative approach that leverages technological advancements and clinical expertise. Ultimately, such efforts hold promise for advancing personalized management strategies and improving outcomes for patients with Dupuytren’s disease.

## Figures and Tables

**Table 1:** Large language models comparative table.

Model Name	Developer	Underlying Architecture	Medical Fine-Tuning	Accessibility	Known Limitations	Time to Generate Response (Approx.)
GPT-4o	OpenAI	GPT-4	General medical capabilities, no specific fine-tuning	Free and Paid (Pro version)	May generate confident but incorrect responses, lacks real-time updates, creates wrong references.	~10–15 s
Gemini 2.0	Google	Gemini	Limited fine-tuning on medical datasets	Free and Paid (Pro version)	Can be inconsistent in complex cases, prone to hallucinations	~8–12 s
Perplexity AI V3	Perplexity AI	Hybrid AI Model	Optimized for research-based queries	Free with query limits	Limited medical validation, relies on web data	~12–18 s
DeepSeek V3	DeepSeek AI	Transformer-based	No medical-specific fine-tuning	Free and Paid	Less robust in specialized domains, lower consistency	~15–20 s
Copilot (GPT-4)	Microsoft	GPT-4	General AI, not specifically trained for medicine	Free with Microsoft 365	Less accurate in medical decision-making, lacks fine-tuning	~10–15 s

**Table 2:** Management Recommendations from Artificial Intelligence and Experienced Hand Surgeons.

Case	Case Description	ChatGPT’s Management	Gemini’s Management	Perplexity’s Management	DeepSeek’s Management	Copilot’s Management	I Surgeon Management	II Surgeon Management	III Surgeon Management
1	54-year-old male, right-hand dominant manual laborer, untreated Dupuytren’s in the 4th ray with a 30° MCP contracture, no comorbidities, non-smoker, no alcohol.	Mild MCP contracture often managed with minimally invasive methods (e.g., PNA or CCH) plus hand therapy to maintain function, especially given manual labor demands.	Minimally invasive: PNA or Cordless Needle Fasciotomy (CNF) followed by hand therapy.	Mild MCP contracture: Minimally invasive methods (PNA or CCH) plus hand therapy, considering manual labor demands.	PNA or collagenase injection for contracture correction. Minimally invasive options are ideal for manual laborers to ensure quick recovery.	Collagenase injections for 30° MCP contracture.	PNA	open fasciectomy	PNA
2	61-year-old male, right-hand dominant IT worker, untreated Dupuytren’s in the 4th ray with 35° MCP and 50° PIP contractures, hypertension, non-smoker, moderate alcohol.	The 50° PIP contracture may require a more interventional approach. Collagenase injection or PNA could be attempted first. If inadequate correction is achieved, consider limited fasciectomy, followed by hand therapy.	Minimally invasive: Collagenase injection or PNA. If inadequate, consider limited fasciectomy followed by hand therapy.	Attempt collagenase injection or PNA first. If inadequate correction, proceed with limited fasciectomy followed by hand therapy.	Collagenase or PNA for MCP and PIP contractures. Collagenase may be preferred for the PIP joint due to its effectiveness	Fasciectomy surgery for 35° MCP and 50° PIP contractures.	PNA	open fasciectomy	Fasciectomy
3	74-year-old female, right-hand dominant, Dupuytren’s in the 4th and 5th rays, 30° MCP and 80° PIP (5th), T2DM/HTN, active smoker, no alcohol. Previous needle aponeurotomy.	Given severe 80° PIP contracture and recurrence despite prior needle aponeurotomy, a more definitive surgical approach—such as limited fasciectomy or dermofasciectomy—may be needed. Emphasize smoking cessation, optimal medical management, and postoperative hand therapy.	Surgical: Limited fasciectomy or dermofasciectomy. Emphasize smoking cessation, optimal medical management, and postoperative hand therapy.	Given severe 80° PIP contracture and recurrence, opt for limited fasciectomy or dermofasciectomy. Emphasize smoking cessation and postoperative hand therapy.	Partial fasciectomy or collagenase for severe PIP contracture. Fasciectomy may provide longer-lasting results given the severity.	Fasciectomy surgery for 30° MCP and 80° PIP (5th ray) contractures.	Limited fasciectomy	PNA	Fasciectomy
4	44-year-old male, right-hand dominant manual laborer, Dupuytren’s with a palpable nodule in the palm but no contracture, no comorbidities, non-smoker, no alcohol.	In the absence of contracture, conservative management (observation, possible night splinting, and/or hand therapy) is appropriate. Intervene only if progression or functional limitation develops.	Conservative: Observation, possible night splinting, and/or hand therapy. Intervene only if progression or functional limitation develops.	Conservative management with observation and possible night splinting. Intervene only if progression occurs.	Monitoring only; no active treatment needed. Educate the patient about disease progression.	Observation and monitoring of the palmar nodule.	Conservative—hand therapy	if nodule painful when gripping tools then CCH if available or localised dermatofasciectomy and small FTSG. If no pain, then conservative.	Conservative
5	45-year-old male, right-hand dominant, mild Dupuytren’s with a 15° MCP contracture, no other digit involvement, no significant medical history, non-smoker, no alcohol.	A mild 15° contracture may not be functionally limiting. Watchful waiting or conservative therapy is often sufficient. If progression occurs or function declines, consider minimally invasive intervention (CCH or PNA).	Conservative: Watchful waiting or conservative therapy. Consider minimally invasive intervention (CCH or PNA) if progression occurs or function declines.	Watchful waiting with conservative therapy. Consider minimally invasive intervention (CCH or PNA) if progression happens.	Conservative monitoring; intervene if progression occurs. Consider PNA or collagenase if contracture worsens.	Collagenase injections for 15° MCP contracture.	PNA	PNA	PNA
6	34-year-old male, right-hand dominant professional guitarist, palpable palmar nodule, no contracture, no comorbidities, non-smoker, no alcohol.	Since there is no contracture, observation is advised. Hand therapy or gentle stretching may help maintain mobility. Interventional treatment can be deferred unless a contracture develops or function is clearly compromised.	Conservative: Observation. Hand therapy or gentle stretching may help maintain mobility. Defer intervention unless contracture develops or function is compromised.	Observation with gentle hand therapy or stretching. Defer interventional treatment.	Monitoring; reassurance and no active treatment. Early intervention may be considered if progression impacts his profession.	Observation and monitoring of the palmar nodule.	Conservative	conservative if asymptomatic. If painful to grip pressure, then PNA.	Conservative
7	79-year-old female, right-hand dominant, untreated Dupuytren’s in the 4th ray with 20° MCP and 50° PIP contracture, previously treated 5th ray with collagenase, HTN, prior MI, asthma, non-smoker, no alcohol.	Moderate PIP involvement (50°). Given past success with collagenase, a repeat minimally invasive procedure (PNA or CCH) could be appropriate. If inadequate or recurrence is severe, consider limited fasciectomy, mindful of comorbidities and age.	Minimally invasive: Repeat PNA or CNF. If inadequate or recurrence is severe, consider limited fasciectomy, mindful of comorbidities and age.	Repeat minimally invasive procedure (PNA or CCH). Consider limited fasciectomy if inadequate, mindful of comorbidities.	Partial fasciectomy for recurrence after collagenase. Surgical intervention is preferred for recurrent cases.	Collagenase injections for 20° MCP and 50° PIP contractures.	Limited fasciectomy	dermatofasciectomy over proximal phalanx, open fasciectomy to pretendinous cord. Ideally under regional block.	Fasciectomy
8	56-year-old male, left-hand dominant pianist, Dupuytren’s in the 4th/5th rays (5th: 40° MCP, 30° PIP; 4th: 10° MCP, 10° PIP), T2DM, occasional alcohol, non-smoker.	A minimally invasive release (CCH or PNA) for the more pronounced 5th ray contracture may preserve hand function essential for piano. If nonsurgical methods fail, limited fasciectomy could be considered. Post-procedure hand therapy is important.	Minimally invasive: CCH or PNA for the 5th ray. If nonsurgical methods fail, consider limited fasciectomy. Post-procedure hand therapy is important.	Minimally invasive release (CCH or PNA) for the 5th ray. Follow with hand therapy to preserve piano function.	Collagenase or PNA for MCP and PIP contractures. Prioritize treatment for the 5th ray to restore hand function for piano playing.	Fasciectomy surgery for 40° MCP and 30° PIP (5th ray), 10° MCP and 10° PIP (4th ray) contractures.	PNA	open fasciectomy	Fasciectomy
9	83-year-old female, right-hand dominant, advanced Dupuytren’s of the 5th ray (30° MCP, 70° PIP), multiple recurrences, T2DM/HTN, non-smoker, no alcohol.	With significant PIP contracture (70°) and multiple recurrences, a more definitive procedure like dermofasciectomy with skin graft may reduce recurrence risk. Surgical risks must be balanced against comorbidities and overall function.	Surgical: Dermofasciectomy with skin graft may reduce recurrence risk. Surgical risks must be balanced against comorbidities and overall function.	Dermofasciectomy with skin graft, balancing surgical risks and overall function.	Total fasciectomy or dermofasciectomy for severe recurrence. Aggressive treatment is needed to address advanced disease.	Fasciectomy surgery for 30° MCP and 70° PIP contractures.	Limited fasciectomy	if the joint is currently dislocated/subluxated- amputation. If the joint is stable, dermatofasciectomy.	Amputation will be discussed but only if it is symptomatic enough
10	86-year-old male, right-hand dominant, severe recurrent Dupuytren’s in multiple rays of both hands, multiple prior surgeries, T2DM, CAD (2 MIs), CKD stage III, AF on anticoagulation, non-smoker, no alcohol. Severe contractures limiting daily activities.	With extensive disease, scarring, and significant comorbidities, additional surgery (e.g., wide fasciectomy/dermofasciectomy) carries higher risk. A multidisciplinary approach is essential. In extreme cases, salvage procedures (including possible digit amputation) may be considered for severely contracted fingers.	Multidisciplinary: A multidisciplinary approach is essential. In extreme cases, salvage procedures (including possible digit amputation) may be considered for severely contracted fingers.	Multidisciplinary approach. Potentially consider salvage procedures, including possible digit amputation in extreme cases.	Palliative PNA or collagenase due to comorbidities. Surgery is high-risk; focus on improving function with minimally invasive options.	Fasciectomy surgery for severe flexion contractures with scarring.	PNA	multidigit PNA	Potential amputation on the LF but other will need dermatofasciectomy
11	74-year-old male, right-hand dominant, advanced recurrent Dupuytren’s in the left 4th finger, 2 prior limited fasciectomies, well-controlled HTN, stage II CKD, T2DM, former smoker, minimal alcohol. Severe flexion contractures with scarring.	Recurrent disease with significant functional impairment and surgical scarring may warrant more radical surgery (e.g., dermofasciectomy) to reduce further recurrence. Comorbidities and patient goals must be considered. Specialized hand therapy and possible arthrodesis if PIP correction is not feasible.	Surgical: Dermofasciectomy may be warranted to reduce further recurrence. Comorbidities and patient goals must be considered. Specialized hand therapy and possible arthrodesis if PIP correction is not feasible.	Dermofasciectomy with specialized hand therapy. Consider arthrodesis if PIP correction is not feasible.	Dermofasciectomy for severe recurrence and scarring. This approach reduces the risk of further recurrence in advanced cases.	Fasciectomy surgery for 40° MCP and 20° PIP contractures.	PNA	dermatofasciectomy	Fasciectomy
12	68-year-old female, right-hand dominant, recurrent Dupuytren’s in the 4th/5th rays, 40° MCP and 20° PIP in the 5th, T2DM, well-controlled HTN, ex-smoker, moderate alcohol. Prior needle aponeurotomy.	With progressive contracture and functional deficits despite prior needle aponeurotomy, consider repeat minimally invasive intervention (CCH or PNA) or limited fasciectomy if needed. Optimize comorbidities and provide supervised hand therapy post-procedure.	Minimally invasive: Repeat CCH or PNA, or limited fasciectomy if needed. Optimize comorbidities and provide supervised hand therapy post-procedure.	Repeat minimally invasive intervention or limited fasciectomy. Optimize comorbidities and provide supervised hand therapy.	Collagenase or partial fasciectomy for recurrence. Collagenase may be effective for moderate contractures.	Fasciectomy surgery for 50° MCP and 90° PIP contractures with marked scarring.	PNA	limited fasciectomy to LF, PNA to other digits.	Fasciectomy
13	74-year-old male, right-hand dominant, advanced recurrent Dupuytren’s of the 5th ray (50° MCP, 90° PIP), prior partial fasciectomy and PNA, T2DM, CKD III, AF on warfarin, ex-smoker, no alcohol. Marked scarring, limited ROM.	Severe flexion contracture and multiple recurrences in a patient with significant comorbidities. A more definitive solution (e.g., dermofasciectomy with graft) may be necessary, though high surgical risk. Tailor intervention to patient’s functional needs, possibly considering less invasive releases if feasible.	Surgical: Dermofasciectomy with graft may be necessary, though high surgical risk. Tailor intervention to patient’s functional needs, possibly considering less invasive releases if feasible.	Dermofasciectomy with graft, carefully considering high surgical risks and patient’s functional needs.	Dermofasciectomy for severe recurrence and limited ROM. Radical treatment is necessary for functional improvement.	Observation and monitoring of nodular thickening without flexion contractures.	limited fasciectomy	dermatofasciectomy but if there is poor perfusion with extension then for amputation	Amputation
14	68-year-old male, right-hand dominant, Dupuytren’s in the palmar fascia of the left hand, T2DM, HTN, CKD II, non-smoker, occasional alcohol, nodular thickening but no flexion contractures.	Without contractures, a conservative approach (observation, hand therapy) is appropriate. Interventional treatment is deferred unless contractures develop and impair function.	Conservative: Observation and hand therapy. Interventional treatment is deferred unless contractures develop and impair function.	Conservative approach with observation and hand therapy.	Monitoring; no active treatment needed. Intervene only if contractures develop.	Fasciectomy surgery for 25° MCP and 10° PIP contractures.	conservative	if dermal nodules are symptomatic, then fasciectomy and full thickness skin graft.	Limited Fasciectomy
15	62-year-old male, left-hand dominant, recurrent Dupuytren’s primarily in the 4th finger, prior partial fasciectomy (6 years ago) and collagenase injection (3 years ago). T2DM, hyperlipidemia, post-CABG, non-smoker, occasional alcohol. Now 25° MCP/10° PIP.	A repeat minimally invasive option (CCH or PNA) may help if the contracture worsens function. If these measures are insufficient, limited fasciectomy could be reconsidered. Emphasize post-procedure therapy to optimize grip and function.	Minimally invasive: Repeat CCH or PNA. If insufficient, limited fasciectomy could be reconsidered. Emphasize post-procedure therapy to optimize grip and function.	Repeat minimally invasive treatment or limited fasciectomy. Emphasize post-procedure therapy.	Repeat fasciectomy or dermofasciectomy for recurrence. Surgical intervention is preferred for recurrent disease.	Collagenase injections for 10° MCP contracture.	PNA	PNA	Fasciectomy
16	52-year-old male, left-hand dominant, early-stage Dupuytren’s in the palm near the ring finger with minimal 10° MCP contracture, no comorbidities, non-smoker, no alcohol, mild functional impact (graphic designer).	With minimal contracture and mild symptoms, observation and conservative measures (e.g., stretching, splinting) are recommended. Intervene only if progression occurs or function significantly declines.	Conservative: Observation and conservative measures (e.g., stretching, splinting) are recommended. Intervene only if progression occurs or function significantly declines.	Observation with conservative measures like stretching and splinting.	Monitoring; intervene if contracture progresses. Consider PNA or collagenase if functional impact increases.	Observation and monitoring of the small palmar nodule with ~5° MCP contracture.	conservative	conservative	PNA
17	49-year-old male, left-hand dominant mechanical engineer, early Dupuytren’s with a small palmar nodule, ~5° MCP contracture, no comorbidities, non-smoker, occasional alcohol, concerned about progression.	Since contracture is minor, a conservative/watchful-waiting approach is appropriate. Provide reassurance, possible night splinting, and routine follow-up to detect changes. If progression compromises function, consider PNA or CCH.	Conservative: Watchful-waiting approach. Provide reassurance, possible night splinting, and routine follow-up to detect changes. If progression compromises function, consider PNA or CCH.	Conservative approach with watchful waiting and routine follow-up.	Monitoring; reassurance and no active treatment. Address patient concerns about progression.	Collagenase injections for 15° MCP contracture.	conservative	Conservative	PNA
18	72-year-old male, right-hand dominant, mild Dupuytren’s in the 3rd/4th rays, 15° MCP contracture at the 5th, HTN on meds, hypercholesterolemia, non-smoker, social alcohol, mild symptoms, frequent typing/writing.	Early-stage disease with minimal functional impact. Monitor progression and maintain ROM (hand therapy). Intervention (PNA or CCH) can be considered if contracture or symptoms worsen.	Conservative: Monitor progression and maintain ROM (hand therapy). Intervention (PNA or CCH) can be considered if contracture or symptoms worsen.	Monitor progression and maintain range of motion with hand therapy. Consider PNA or CCH if worsening.	Monitoring; intervene if contracture progresses. Consider treatment if symptoms worsen.	Observation and monitoring of untreated Dupuytren’s in the 4th/5th rays.	PNA	PNA	PNA
19	59-year-old male, right-hand dominant, untreated Dupuytren’s in the 4th/5th rays of the left hand (5th: 50° MCP, 50° PIP; 4th: 30° MCP, 30° PIP). Carpenter with T2DM, mild HTN, non-smoker, social alcohol, progressive contractures over 2 years.	Given progressive contractures that impact work-related dexterity, start with minimally invasive methods (CCH or PNA). If inadequate, proceed to limited fasciectomy on the most affected fingers. Post-procedure therapy is crucial for occupational demands.	Minimally invasive: CCH or PNA. If inadequate, proceed to limited fasciectomy on the most affected fingers. Post-procedure therapy is crucial for occupational demands.	Start with minimally invasive methods. Proceed to limited fasciectomy on most affected fingers if needed.	Collagenase or partial fasciectomy for progressive contractures. Early intervention is recommended to prevent further functional loss.	Fasciectomy surgery for 50° MCP and 50° PIP (5th ray), 30° MCP and 30° PIP (4th ray) contractures.	PNA	PNA	Fasciectomy
20	68-year-old male, right-hand dominant, moderate Dupuytren’s of the left 5th ray, 40° MCP and 70° PIP, well-controlled hypertension, non-smoker, social alcohol, physically active, progressive over 3 years with functional impairment.	Given progressive functional impairment and a significant PIP contracture (70°), a minimally invasive approach (CCH or PNA) could be attempted first. If insufficient, consider limited fasciectomy. Post-procedure therapy is recommended to optimize range of motion and minimize disruption to daily activities.	Minimally invasive: CCH or PNA could be attempted first. If insufficient, consider limited fasciectomy. Post-procedure therapy is recommended to optimize range of motion and minimize disruption to daily activities.	Attempt minimally invasive approach first. Consider limited fasciectomy if insufficient. Recommend post-procedure therapy.	Partial fasciectomy or collagenase for functional impairment. Prioritize treatment to restore hand function.	Fasciectomy surgery for 40° MCP and 70° PIP contractures.	limited fasciectomy	dermatofasciectomy and FTSG	Amputation
21	68-year-old male, left-hand dominant, severe recurrent Dupuytren’s of the left 4th finger (60° MCP, 80° PIP), prior PNA 3 years ago with temporary relief, well-controlled HTN, non-smoker, social alcohol, progressive over 2 years, reduced grip.	With severe recurrence and functional limitation, repeated needle aponeurotomy or collagenase might be only temporary. A more definitive procedure (limited fasciectomy or dermofasciectomy) could offer more durable correction. Postoperative therapy is crucial to maintain gains and reduce recurrence.	Surgical: Limited fasciectomy or dermofasciectomy could offer more durable correction. Postoperative therapy is crucial to maintain gains and reduce recurrence.	Limited fasciectomy or dermofasciectomy with crucial postoperative therapy.	Dermofasciectomy for severe recurrence and reduced grip. Aggressive treatment is needed for functional improvement.	Fasciectomy surgery for 60° MCP and 80° PIP contractures.	limited facsiectomy	open fasciectomy	Fasciectomy
22	50-year-old male, right-hand dominant, Dupuytren’s primarily in the left 4th ray, 25° MCP contracture, carpenter with no comorbidities, non-smoker, no alcohol, progressive over 2 years, mild functional impact.	With a 25° MCP contracture and progressive changes impacting grip, a minimally invasive approach (CCH or PNA) may help preserve function. If mild contracture is not severely affecting tasks, conservative observation is acceptable until progression justifies intervention.	Minimally invasive: CCH or PNA may help preserve function. If mild contracture is not severely affecting tasks, conservative observation is acceptable until progression justifies intervention.	Minimally invasive approach (CCH or PNA) to preserve function. Conservative observation acceptable if involvement is mild.	Collagenase or PNA for functional impact. Minimally invasive options are ideal for early intervention.	Collagenase injections for 25° MCP contracture.	PNA	Open fasciectomy if the patient can take time off work. PNA if limited time off work.	Limited Fasciectomy

Abbreviation: MCP = Metacarpophalangeal joint; PIP = Proximal interphalangeal joint; PNA = Percutaneous needle aponeurotomy; CCH = Collagenase Clostridium histolyticum (e.g., Xiaflex); T2DM = Type-2 diabetes mellitus; CKD = Chronic kidney disease; HTN = Hypertension; MI = Myocardial infarction; AF = Atrial fibrillation; CABG = Coronary artery bypass graft.

**Table 3:** Coded Representation of Management Modalities Selected by LLMs and Surgeons for Analytical Comparison.

Case	ChatGPT’s Management	Gemini’s Management	Perplexity’s Management	DeepSeek’s Management	Copilot’s Management	I Surgeon Management WR	II Surgeon Management RR	III Surgeon Management SN
1	1/2	1/3	1/2	1/2	2	1	7	1
2	1/2/4	1/2/4	1/2/4	1/2	4	1	7	7
3	4/5	4/5	4/5	7/2	4	4	1	7
4	8	8	8	8	8	8	2/6/8	8
5	8	8	8	1/2	2	1	1	1
6	8	8	8	8	8	8	8/1	8
7	1/2/4	1/2/4	1/2/4	7	2	4	5/7	7
8	2/1/4	2/1/4	2/1	4	4	1	7	7
9	6	6	5/6	4	4	4	10/5	10
10	9/10	9/10	1/2	4	4	1	1	10/5
11	5/11	5/11	5	4	4	1	5	7
12	1/2/4	1/2/4	2/7	4	4	1	4/1	7
13	6	6	5	8	8	4	5/10	10
14	8	8	8	4	4	8	6	4
15	1/2/4	1/2/4	4/5	2	2	1	1	7
16	8	8	8	1/2	8	8	8	1
17	8	8	8	2	2	8	8	1
18	8	1/2	8	1/2	8	1	1	1
19	1/2/4	1/2/4	2/7	4	4	1	1	7
20	1/2/4	1/2/4	7/2	4	4	4	6	10
21	4/5	4/5	5	4	4	4	7	7
22	1/2	1/2	1/2	2	2	1	7/1	4

The legend for the numerical codes is as follows: 1—represents Percutaneous Needle Aponeurotomy (PNA), 2—stands for Collagenase injection (CCH), 3—indicates Cordless Needle Fasciotomy (CNF), 4—refers to Limited fasciectomy, 5—denotes Dermofasciectomy, 6—signifies Dermofasciectomy with skin graft, 7—corresponds to Partial fasciectomy, 8—represents Conservative management (observation, splinting, or hand therapy), 9—indicates a Multidisciplinary approach, 10—refers to Salvage procedures (including possible digit amputation), and 11—stands for Arthrodesis.

**Table 4:** Extended Performance Metrics of LLMs and Agreement with Surgeons.

Metric	ChatGPT	Gemini	Perplexity	DeepSeek	Copilot
Union Accuracy (%)	81.8	86.4	77.3	63.6	40.9
Surgeon I Agreement (%)	72.7	77.3	54.5	45.5	31.8
Surgeon II Agreement (%)	36.4	36.4	40.9	27.3	18.2
Surgeon III Agreement (%)	18.2	22.7	40.9	27.3	31.8
Average Surgeon Agreement (%)	42.4	45.5	45.4	33.4	27.3
Avg Options Count	2.0	2.1	2.0	1.8	1.3
Avg Precision (%)	62.0	64.0	60.0	55.0	45.0
Avg Recall (%)	58.0	60.0	57.0	52.0	42.0
Avg F1 Score (%)	60.0	62.0	58.5	53.5	43.5

**Table 5:** Inter-AI LLMs Agreement.

AI Pair	Agreement (%)
ChatGPT and Gemini	75.0
ChatGPT and Perplexity	70.0
ChatGPT and DeepSeek	65.0
ChatGPT and Copilot	50.0
Gemini and Perplexity	78.0
Gemini and DeepSeek	70.0
Gemini and Copilot	55.0
Perplexity and DeepSeek	68.0
Perplexity and Copilot	52.0
DeepSeek and Copilot	48.0

## Data Availability

Data for this study are available upon reasonable request from the corresponding author.

## Abbreviations

The following abbreviations are used in this manuscript:

AI	Artificial Intelligence
MCP	Metacarpophalangeal Joint
PIP	Proximal Interphalangeal Joint
PNA	Percutaneous Needle Aponeurotomy
CCH	Collagenase Clostridium Histolyticum (e.g., Xiaflex)
TPED	Total Passive Extension Deficit
T2DM	Type 2 Diabetes Mellitus
CKD	Chronic Kidney Disease
HTN	Hypertension
MI	Myocardial Infarction
AF	Atrial Fibrillation
CABG	Coronary Artery Bypass Graft
